# The Cac2 subunit is essential for productive histone binding and nucleosome assembly in CAF-1

**DOI:** 10.1038/srep46274

**Published:** 2017-04-18

**Authors:** Francesca Mattiroli, Yajie Gu, Jeremy L. Balsbaugh, Natalie G. Ahn, Karolin Luger

**Affiliations:** 1 Howard Hughes Medical Institute, University of Colorado Boulder, Department of Chemistry and Biochemistry, Boulder, CO 80303, USA; 2 University of Colorado at Boulder, Department of Chemistry and Biochemistry, Boulder, CO 80303, USA; 3 Colorado State University, Department of Biochemistry and Molecular Biology, Fort Collins, CO 80523, USA; 4 University of Colorado at Boulder, Biofrontiers Institute, Department of Chemistry and Biochemistry, Boulder, CO 80303, USA; 5 Institute for Genome Architecture and Function, Colorado State University, Fort Collins, CO 80523, USA

## Abstract

Nucleosome assembly following DNA replication controls epigenome maintenance and genome integrity. Chromatin assembly factor 1 (CAF-1) is the histone chaperone responsible for histone (H3-H4)_2_ deposition following DNA synthesis. Structural and functional details for this chaperone complex and its interaction with histones are slowly emerging. Using hydrogen-deuterium exchange coupled to mass spectrometry, combined with *in vitro* and *in vivo* mutagenesis studies, we identified the regions involved in the direct interaction between the yeast CAF-1 subunits, and mapped the CAF-1 domains responsible for H3-H4 binding. The large subunit, Cac1 organizes the assembly of CAF-1. Strikingly, H3-H4 binding is mediated by a composite interface, shaped by Cac1-bound Cac2 and the Cac1 acidic region. Cac2 is indispensable for productive histone binding, while deletion of Cac3 has only moderate effects on H3-H4 binding and nucleosome assembly. These results define direct structural roles for yeast CAF-1 subunits and uncover a previously unknown critical function of the middle subunit in CAF-1.

Nucleosomes are the primary units of chromatin responsible for the organization of DNA and storage of epigenetic information. The interaction between positively charged histones and negatively charged DNA must be tightly regulated *in vivo* to avoid the formation of non-specific histone·DNA complexes^1^. Histone chaperones guard histones by shielding their charges and controlling their deposition onto DNA^2^. This places histone chaperones at a central position in all pathways that require access to the genome.

During DNA replication, the concerted action of a number of histone chaperones coordinates the recycling of parental histones and the deposition of newly-synthesized histones to maintain nucleosome density and epigenetic information^3^. Chromatin Assembly factor 1 (CAF-1) is a heterotrimeric histone chaperone that mediates the final step in this cascade by assembling nucleosomes following DNA synthesis^4^,^5^,^6^,^7^,^8^. CAF-1 directly interacts with the processivity factor for DNA polymerases, PCNA, which localizes to sites of DNA synthesis during replication and repair^9^,^10^,^11^,^12^,^13^,^14^. The unique role of CAF-1 in chromatin assembly following DNA synthesis makes it essential for cell survival in multicellular organisms^15^,^16^,^17^,^18^. In yeast, deletion of either CAF-1 subunit results in cell viability but affects transcriptional silencing and DNA damage sensitivity^8^.

The structural organization of the CAF-1 complex is only beginning to emerge^19^,^20^, and the direct contribution of each subunit to CAF-1 functions remain unclear. Histone binding by CAF-1 has been attributed to the Cac1 acidic region^6^,^20^, a ~50 amino acids stretch, primarily composed of Glu and Asp residues. Other studies have attributed this function to the small subunit (Cac3 in yeast), as the human and fly homologues bind histones H3 and H4 when tested in isolation^21^,^22^,^23^,^24^. Notably, this subunit is shared with other chromatin-related complexes, such as histone deacetylase complexes, NuRD, NURF and PRC2^25^,^26^,^27^,^28^,^29^. Nonetheless, the direct contribution of this subunit to histone binding in the context of the full CAF-1 complex has not yet been tested. In cells, histones are handed off to CAF-1 by the histone chaperone ASF1, which directly interacts with the C-terminal region of the CAF-1 middle subunit^30^,^31^,^32^,^33^, yet beyond that, the role of the middle subunit for CAF-1 activity remain elusive.

Here we focus on the yeast CAF-1 complex and identify the regions that mediate complex formation between the three CAF-1 subunits (Cac1, Cac2 and Cac3). We show that these interfaces are relevant for CAF-1 function *in vivo*. We find that when bound to Cac1, Cac2 is indispensable for productive interaction with the histones, thus their deposition. The Cac1 acidic region contributes to histone binding and together, these shape a composite interface on CAF-1 for H3-H4 binding.

## Results

### CAF-1 is an elongated complex

**Figure 1:**
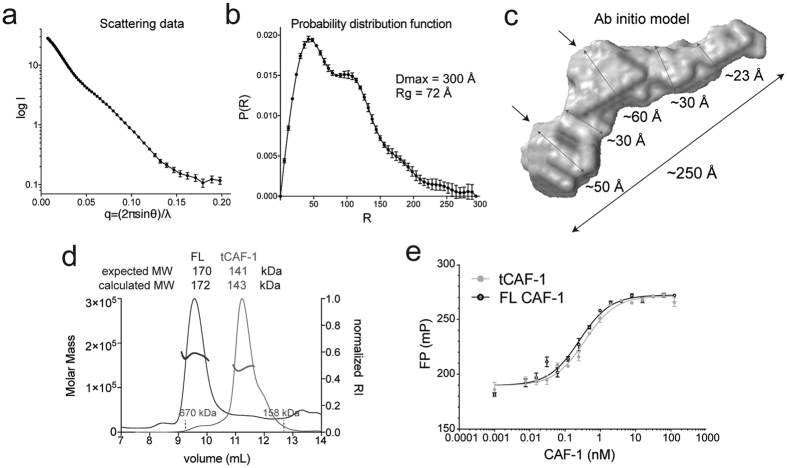
CAF-1 is an elongated complex. (**a**) SAXS scattering data for the full length CAF-1 complex. (**b**) Probability distribution function from the SAXS data. (**c**) Calculated ab initio model from SAXS data. Arrows indicate putative locations of Cac2 and Cac3, respectively, also informed by mass spectrometry. (**d**) SEC-MALS traces of FL CAF-1 and tCAF-1. The protein/DNA elution traces (refractive index, RI) refer to the right y axis, the measured molar mass data points refer to the left y axis. (**e**) Fluorescence polarization measurement of the affinity of FL CAF-1 and tCAF-1 for Alexa488-labeled H3-H4.

Recombinant full-length (FL) yeast CAF-1 complex was expressed in SF21 cells using the MultiBac system^34^. We used small angle X-ray scattering in line with size exclusion chromatography (SEC-SAXS) to investigate the architecture of the complex (Fig. 1a–c). Previous data showed that the three CAF-1 subunits, Cac1, Cac2 and Cac3, assemble in a 1:1:1 stoichiometry^34^, which was confirmed by our analysis with size exclusion chromatography in line with multi-angle light scattering (SEC-MALS) (Fig. 1d). The SAXS probability function (P(r)) displays a bimodal distribution indicative of two structural units connected by a less organized linker, with an extended maximum distance for the entire complex of 250–300 Å (Fig. 1b). This can be visualized in the ab initio model generated from the SEC-SAXS data (Fig. 1c). An extended conformation of the complex is also suggested by SEC-MALS, where CAF-1 elutes from the column much earlier than expected for a globular complex with similar molecular weight (Fig. 1d). Based on their expected size, we speculate that the two lobes in the *ab initio* model identify the location of the two WD40 subunits, Cac2 and Cac3 (Fig. 1c, arrows), while Cac1 is likely responsible for the elongated shape.

### Cac1 mediates CAF-1 complex assembly

Previous work showed that the middle subunit of CAF-1 interacts with the C-terminal region of the large subunit (Cac2 and Cac1 respectively in yeast)^6^. We determined through proteolysis experiments that Cac3 interacts with the central portion of Cac1 (from residue 233; Supplementary Figure 1). Based on this information, we engineered a Cac1 fragment starting at residue 233 and extending to the very C-terminal amino acid 606 (named tCac1 for truncated Cac1), to obtain a minimal Cac1 construct still able to form a complex with the other CAF-1 subunits. Indeed, tCac1 formed a trimeric complex together with Cac2 and Cac3, which we name tCAF-1. This complex maintains a 1:1:1 stoichiometry (Fig. 1d) and binds histones H3-H4 with the same affinity as FL CAF-1 (Fig. 1e and Table 1), demonstrating that tCAF-1 retains the molecular requirements for CAF-1 trimer assembly and histone binding.

**Figure 2:**
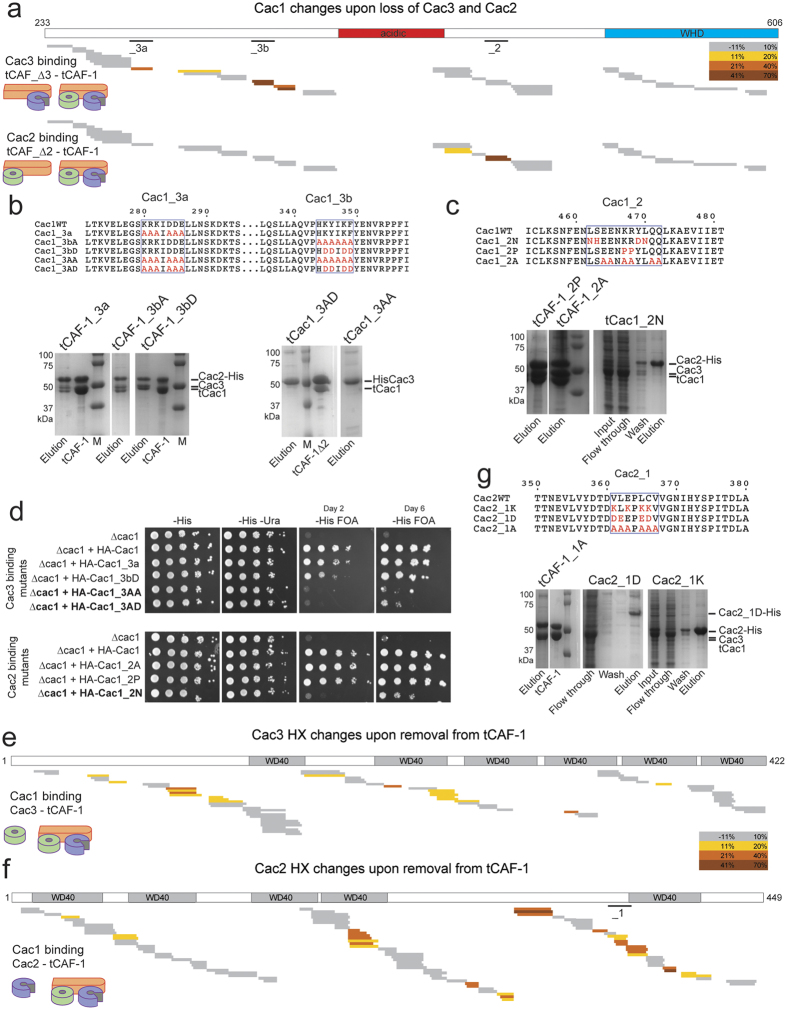
Cac1 mediates CAF-1 complex formation. (**a**) HX-MS heatmap of the differences in deuteron uptake at 60 minutes in Cac1, when Cac3 or Cac2 are omitted from the sample (comparison between tCAF_∆3 or tCAF_∆2 respectively). The difference was calculated as percent uptake in the subcomplex (unbound form) minus the percent uptake in tCAF-1 (bound form). (**b**) Alignment of the Cac1 regions interacting with Cac3, and the mutants designed and analyzed in this study. SDS PAGE of the His-pulldown from insect cells using the Cac1_3a, Cac1_3b or the Cac1_3 mutants (mutated both on peptide a and b). Cac2-His was used as a bait in the left panels. His-Cac3 was used as a bait in the right panels (Cac2 was not present in these samples). Full-length gels are shown in Supplementary Figure 4a. (**c**) Alignment of the Cac1 regions interacting with Cac2 and the mutants designed in this study. SDS PAGE of the His-pulldown from insect cells using the Cac1_2 mutant proteins. Cac2-His was used as a bait. Full-length gels are shown in Supplementary Figure 4b. (**d**) Silencing assay performed with yeast strains expressing the Cac1 mutations at the_3a,_3b or_2 sites. Samples were spotted at 0–10^1^–10^2^–10^3^–10^4^ dilutions from a OD600 = 1 stock. (**e**) HX-MS heatmap of the differences in deuteron uptake at 60 minutes in Cac3, when bound in tCAF-1. The difference was calculated as percent uptake in the single subunit (unbound form) minus the percent uptake in tCAF-1 (bound form). WD40 domain mapping in the schematics is based on the Uniprot prediction. (**f**) HX-MS heatmap of the differences in deuteron uptake at 60 minutes for Cac2 when it’s removed from tCAF-1. The difference was calculated as in panel **e** but related to Cac2. WD40 domain mapping in the schematics is based on the Uniprot prediction._1 depicts the peptide used for the mutagenesis studies shown in panel g. (**g**) Alignment of the Cac2 region interacting with Cac1 and the mutants designed. SDS PAGE of the His-pulldown from insect cells using the Cac2_1 mutants as bait. Full-length gels are shown in Supplementary Figure 4c. Supplementary File 1 contains all the HX uptake values for the experiments shown in this figure.

To identify the peptides involved in mediating the interactions between CAF-1 subunits, we applied hydrogen-deuterium exchange coupled to mass spectrometry (HX-MS) to the tCAF-1 complex and its sub-complexes lacking either the middle subunit Cac2 (tCAF_∆2) or the small subunit Cac3 (tCAF_∆3), as well as to the Cac2 and Cac3 subunits in isolation. Notably, all of these complexes could be purified using standard protocols and eluted as well-behaved monodisperse complexes of the expected size and stoichiometry in gel filtration (Supplemetary Figure 2a). HX-MS can be used to identify binding interfaces and conformational changes in protein complexes due to the specific protection from exposure to deuterated solvents as a consequence of protein-protein interactions^35^. From the HX-MS experiments, we obtained information on about 110–120 peptides from each subunit per sample, with a protein coverage of 90% for Cac1, 94% for Cac2 and 95% for Cac3. For comparative purposes we then focused only on peptides that were present in all samples, bringing the protein coverage to 82% for Cac1, 89% for Cac2 and 85% for Cac3.

To identify the Cac1 regions responsible for Cac3 and Cac2 binding, we compared deuteron uptake of Cac1 peptides within the entire tCAF-1 complex to uptake in the tCAF_∆3 or tCAF_∆2 complex, respectively (Fig. 2a and Supplemetary Figure 2b). Strikingly, upon omission of either subunit, a clear increase in deuteron uptake is seen in distinct regions of tCac1. Cac3 binding to Cac1 results in protection on residues 275–390 in Cac1, while Cac2 binding protects a much shorter region on Cac1 (aa 436–475) (Fig. 2a and Supplemetary Figure 2b). These two regions are separated by the Cac1 acidic domain (aa 383–436), for which we observed low coverage in our HX-MS analysis (Fig. 2a).

Based on these results, we designed charge neutralization and reversal mutations that could abrogate individual CAF-1 subunit interactions *in vitro*. We found that two peptides on Cac1 (peptide 280–286 and 344–349) contact Cac3, and either site is sufficient to support Cac1 interaction with Cac3, as shown by pulldown assays from insect cell lysates expressing either wild type or mutant proteins (Fig. 2b). Cac1-Cac3 interaction was abolished by mutating both regions (tCac1_3AA and tCac1_3AD), but not by the mutation on a single peptide (tCac1_3a and tCac1_3bA or tCac1_3bD) (Fig. 2b). With respect to Cac2, a charged mutation on Cac1 residues 463–475 (tCac1_2 N) abolishes the interaction with Cac1, demonstrating the involvement of this region in binding Cac2 (Fig. 2c).

We tested these Cac1 mutations *in vivo*, by monitoring the transcriptional state of a sub-telomeric reporter *URA3* gene, that is dependent on CAF-1 integrity^8^. In the absence of Cac1, growth on FOA-containing plates is inhibited due to the aberrant expression of the reporter gene, a phenotype that can be rescued by ectopic expression of the wild type FL *CAC1* subunit (Fig. 2d). Strikingly, Cac1 mutants that disrupt binding to Cac2 or Cac3 in the *in vitro* pull-down experiments, show an abnormal growth in this assay, consistent with aberrant CAF-1 functions (Fig. 2d), while mutants that are still able to bind Cac2 and Cac3 *in vitro* have no phenotype and fully rescue the *CAC1* deletion (Fig. 2d). Normal levels of expression of all mutant proteins in yeast was demonstrated by immunoblot (Supplemetary Figure 2c). These data show that interfering with CAF-1 subunit interactions *in vivo* leads to dysfunctional CAF-1 complexes. Importantly, in these *CAC1* mutant strains, the Cac2 and Cac3 protein levels are not directly affected, but rather the proteins are selectively removed from CAF-1. This implies that Cac3 still may function normally in the other complexes that it participates in^25^,^26^,^27^,^28^,^29^.

Cac2 and Cac3, which are predicted to have a typical WD40 fold, both undergo extensive changes in HX when part of the tCAF-1 complex (Fig. 2e,f), suggesting that their overall conformation is affected upon complex formation. To pinpoint the regions involved in direct binding we focused on peptides that show an almost complete protection (deuteron uptake close to 0%) in the bound form, similar to the peptides we have identified on Cac1 (Supplemetary Figure 2b). The complete lack of deuteration in the bound form for these peptides suggests a binding site that is comprised of essentially the entire observed amino acid sequence. We identified a number of peptides that filled these criteria in Cac2, but differences observed in Cac3 were much smaller making it difficult to predict direct binding interfaces. We therefore designed mutations only on Cac2, based on the observed HX changes. Charged mutations of residues 361–367 (named Cac2_1) lead to loss of binding to tCac1, without affecting Cac2 expression (Fig. 2g). Interestingly, in a homology model of Cac2, this region is predicted to be exposed (Supplemetary Figure 2d), supporting the notion that it may directly mediate binding to Cac1. Mutations at other peptides on Cac2 that show significant HX changes disrupted protein expression and hence could not be directly tested for their involvement into binding Cac1. These results support the idea that the Cac1-Cac2 interaction occurs between residues 462–472 on Cac1 and residues 361–367 on Cac2, primarily via hydrophobic interactions.

Only very subtle HX changes are detected when probing the Cac2-Cac3 interface within tCAF-1 (Supplemetary Figure 2e), suggesting that these two subunits may not contact each other when bound to Cac1.

Overall, using HX-MS combined with analyses of complex formation in yeast, we have identified regions on each subunit mediating direct interactions between Cac1, Cac2 and Cac3. We show that Cac1 mediates all key interactions that hold the CAF-1 trimer together, supporting previous *in vitro* reconstitutions and recent crosslinking data^6^,^19^,^20^,^36^. We have identified mutations in the Cac1 and Cac2 subunit that do not affect expression or folding of the mutant subunits, yet disrupt complex formation and compromise CAF-1 functions *in vivo*, and therefore can be used to directly investigate CAF-1 subunit contributions in cells without the need of depleting the proteins in the nucleus. This is particularly important to study the role of Cac3 *in vivo*, because it also participates in other complexes outside of the CAF-1 complex.

### Cac3 is dispensable for H3-H4 binding in CAF-1, while Cac2 is required

**Figure 3:**
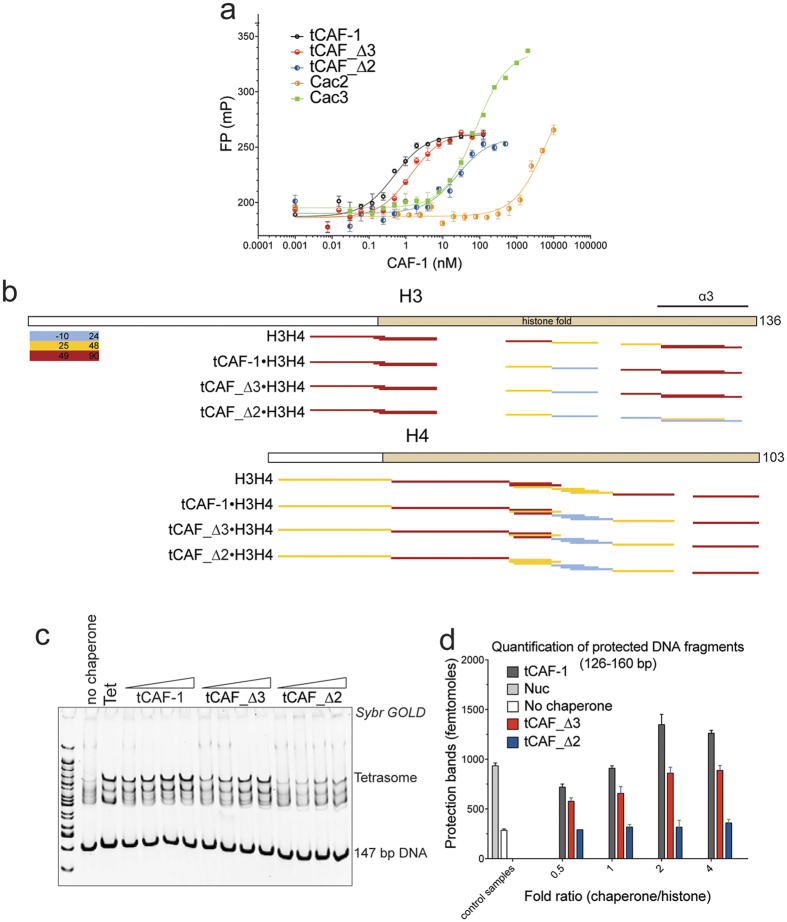
Cac2 is essential for proper histone binding in CAF-1. (**a**) Affinity measurements (fluorescence polarization) of CAF-1 subunits and sub-complexes to H3-H4 labeled with Alexa488. Values are reported in Table 1. (**b**) HX-MS heatmap of deuteron uptake for histone H3 and H4 peptide (histone alone or in complex with different CAF-1 substrates). The deuterium update percentage for each peptide was calculated and scored. The results for tCAF-1 are published in Ref. 37 Supplementary File 2 contains all the HX uptake values for H3-H4 shown in this figure panel. (**c**) Tetrasome assembly assay with 147 bp 601 DNA. Products were resolved on a 6% native gel. (H3-H4)_2_ tetramer concentration is 100 nM, chaperones are titrated with 0.5, 1, 2 and 4 fold over tetramer concentration, DNA is 100 nM. (**d**) Quantification of the nucleosome bands (126–160 bp) from the NAQ assays. The nucleosome assembly reactions on 207 bp DNA were treated with MNase. The DNA was then purified and quantified from Bioanalyzer runs. Mean ± SEM is shown.

We next wanted to identify the CAF-1 regions involved in histone binding. To this end, we used the tCAF-1 subcomplexes and deletion constructs in H3-H4 binding assays. Cac3 in isolation is able to bind H3-H4, as was observed for its metazoan homologues^21^,^22^,^23^. However, its affinity for H3-H4 is over 50-fold weaker than that of the trimeric tCAF-1 complex (Fig. 3a and Table 1). We therefore tested whether Cac3 was required for histone binding in the context of CAF-1. Strikingly, removal of Cac3 only had a minor effect on H3-H4 binding affinity, while the omission of Cac2 resulted in a 50-fold decrease of binding affinity, even though it has no affinity for histones when tested in isolation (Fig. 3a and Table 1). This demonstrates that Cac2, together with Cac1, is important to shape the H3-H4 binding interface on CAF-1, with very little contribution from Cac3.

The contributions of Cac2, but not of Cac3, to H3-H4 binding are surprising in light of previous observations suggesting that Cac3 coordinates histone binding^21^,^22^,^23^,^24^. To further validate these findings, we investigated how these subunits contribute to histone binding using HX-MS. We decided to focus on HX changes in the histone proteins themselves to investigate how their accessibility to solvent is affected in absence of Cac2 or Cac3, as analysis of the tCAF-1 subunits shows only subtle and distributed changes overall^20^,^37^. We have shown that the trimeric tCAF-1 complex stabilizes the histone fold core of WTH3-H4, but does not interfere with the tetramerization of the histones, as no significant changes are seen in the C-terminal part of H3^37^. Here we show, in line with binding affinity data reported in Fig. 3a, that Cac3 removal appears to have a minimal effect on the protection pattern of histones H3-H4 (Fig. 3b) when comparing the maximum deuteron uptake at 60 minutes for all histone peptides. In fact, the HX profiles of WTH3-H4 are very similar when bound to tCAF-1 or tCAF_∆3, suggesting only a minor role, if any, for Cac3 in organizing H3-H4 binding in CAF-1. In contrast, in the absence of Cac2, we observe significant differences in the HX protection profile of H3-H4, compared to the HX profile of H3-H4 in complex with trimeric tCAF-1 (Fig. 3b). In this complex (tCAF_Δ2), we note significant protection of the H3 α3 helix (H3 aa 110–126), the region responsible for (H3-H4)_2_ tetramerization (Fig. 3b). This region remains exposed when the histones are in complex with functional tCAF-1 and tCAF-1 lacking Cac3 (tCAF_∆3) (Fig. 3b). This suggests that in absence of Cac2, histones are bound in a different manner, supporting a pivotal role for Cac2 in organizing histone binding by CAF-1.

To understand whether this residual binding of tCAF_Δ2 to histones is sufficient to sustain nucleosome assembly by CAF-1, we performed a tetrasome assembly assay. Here, we observed no product formation in absence of Cac2 (Fig. 3c). This was confirmed when we measured nucleosome assembly activity of the tCAF-1 subcomplexes in a quantitative nucleosome assembly (NAQ) assay^37^ (Fig. 3d). In this assay, we quantify the amount of assembled nucleosomes by treating assembly reactions with Micrococcal Nuclease (MNase), followed by quantification of length and amount of the purified DNA on a Bioanalyzer. These data demonstrate that Cac2 is indispensable for proper histone deposition onto DNA.

Importantly, although Cac3 has no significant effect on histone binding, its depletion has a minor effect on tetrasome and nucleosome formation (Fig. 3c,d), suggesting that this subunit may contribute to the deposition process, possibly indirectly by organizing the Cac1 central region.

### CAF-1 binds H3-H4 through a composite interface

**Figure 4:**
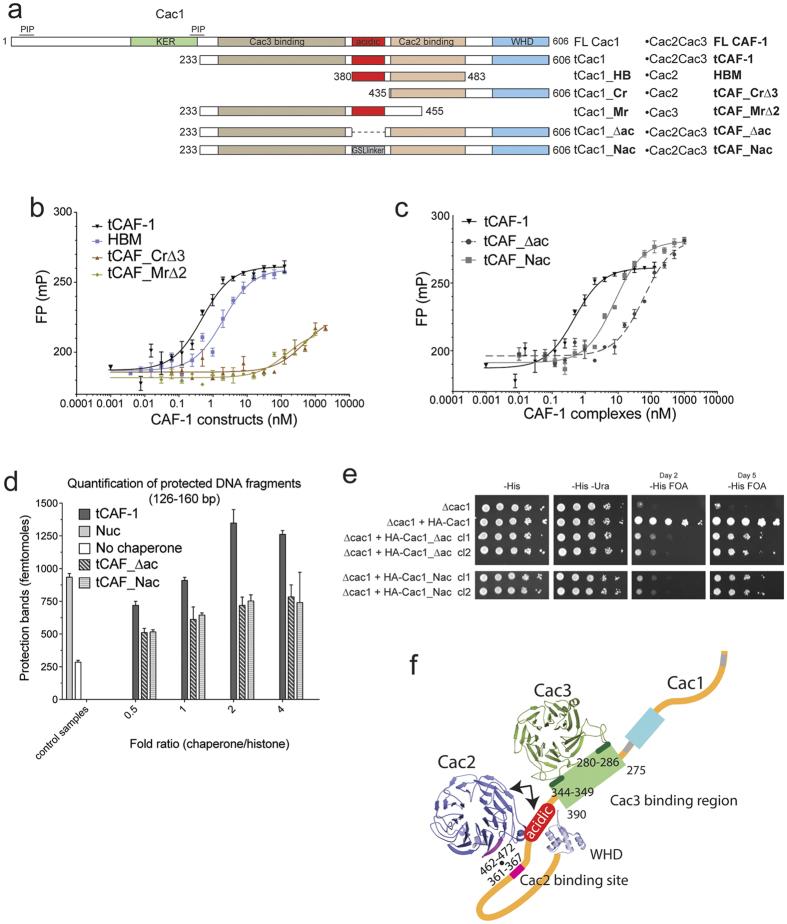
CAF1 binds histones H3-H4 through a composite interface. (**a**) Schematic overview of the constructs used to validate histone binding and deposition mechanism. Summary of the binding affinity and the activities measured for each complex. WHD: winged-helix domain. KER : KER-rich (highly charged, pI = 9.89) domain. PIP: PCNA interaction peptide. HBM: histone binding module. Cr: C-terminal region of Cac1. Mr: middle region of Cac1. ∆ac: tCac1 with deleted acidic region. Nac: tCac1 with neutralized acidic region. (**b**) Affinity measurements (fluorescence polarization) of CAF-1 subunits and sub-complexes described in Fig. 4a, to H3-H4 labeled with Alexa488. Values are reported in Table 1. (**c**) Affinity measurements (fluorescence polarization) of tCAF-1, tCAF_∆ac and tCAF_Nac, to H3-H4 labeled with Alexa488. Values are reported in Table 1. (**d**) Quantification of the nucleosome bands (126–160 bp) from the NAQ assays of tCAF-1, tCAF_∆ac and tCAF_Nac. The nucleosome assembly reactions on 207 bp DNA were treated with MNase. The DNA was then purified and quantified from Bioanalyzer runs. Mean ± SEM is shown of at least two independent experiments. (**e**) Silencing assay performed with yeast strains expressing the Cac1 mutations at the acidic region. Samples were spotted at 0-10^1^-10^2^-10^3^-10^4^ dilutions from a OD600 = 1 stock. (**f**) A yeast CAF-1 model showing the interfaces between the 3 subunits. Histone binding occurs through a composite histone binding interface on CAF-1, including the acidic region of Cac1 and Cac2 (arrows). Data from SEC-SAXS and HX-MS were used to build this structural model. The Cac2 and Cac3 homology models were generated using Phyre2.

Because the acidic region on Cac1 is located in close proximity to the Cac2 binding site mapped by HX-MS (Fig. 2a), and thought to be involved in H3-H4 binding^6^,^20^, we wanted to test if it forms a composite binding site for histones together with Cac2. We first asked whether the acidic region on Cac1, together with Cac1-bound Cac2, constitutes the minimal unit for high affinity H3-H4 binding. Indeed, a complex containing Cac2 and a Cac1 fragment encompassing only the acidic and the Cac2 binding region (tCac1_HB, Fig. 4a) binds H3-H4 with a Kd of ~2 nM, which is within the same range as the affinities measured for CAF-1. We conclude that this complex represents a minimal histone binding module (HBM, Fig. 4b and Table 1), sufficient for high affinity histone binding. When we use a Cac1 construct spanning the C-terminal region (Cr) but lacking the acidic region (tCAF_Cr∆3, Fig. 4a) we lose H3-H4 binding (Fig. 4b and Table 1). Similarly, H3-H4 binding is lost in a Cac1 construct containing the middle region (Mr), including the acidic patch but lacking the Cac2 binding site (tCAF_Mr∆2, Fig. 4a,b and Table 1). Finally, we prepared trimeric tCAF-1 complexes where we either deleted the acidic region in Cac1 (tCAF_∆ac) or replaced it with a neutral Gly-Ser-Leu peptide of the same length (tCAF_Nac) (Fig. 4a). tCAF_∆ac had a markedly reduced affinity for H3-H4 (Kd = 60 nM, Fig. 4c and Table 1), while tCAF_Nac had a less pronounced effect (Kd = 8 nM, Fig. 4c and Table 1). These data demonstrate that the charges in the Cac1 acidic region are important for high affinity histone binding, yet histone binding affinity could be partially rescued by replacing this region with a neutral spacer. These data indicate that the acidic region not only functions through its charged nature, but that it also may exert a structural role in shaping the relative orientation of the Cac2 and the Cac3 binding regions on Cac1.

Notably, a minor effect in nucleosome assembly is seen with these mutants in the NAQ assay (Fig. 4d), indicating that the reduced binding affinity (~10 fold) does not completely preclude CAF-1 function *in vitro*. Nonetheless, when testing these mutations for their transcriptional silencing effect in cells, we observed delayed growth on FOA plates, consistent with a defect in proper CAF-1 function (Fig. 4e and Supplementary Figure 5).

Together these data indicate that the acidic region contributes to the histone binding interface together with Cac1-bound Cac2, and its integrity is important for CAF-1 function in cells. Notably, the more severe effects observed in the NAQ assay in absence of Cac2 (tCAF_Δ2, Fig. 3c) suggest that in the context of the CAF-1 complex, Cac2 is the cornerstone of the CAF-1·histones binding interface.

## Discussion

In this study, we show that CAF-1 has an elongated shape in solution, and that the extended large subunit Cac1 bridges Cac2 and Cac3, which themselves do not interact with each other. By using HX-MS followed by site directed mutagenesis, we identified peptides that form the interfaces between the three subunits of CAF-1 (Fig. 4f). Cac3 binds to the Cac1 middle domain N-terminally to the acidic region, through two main points of contact on Cac1, and Cac2 interacts through a single hydrophobic interaction with the Cac1 C-terminal region located between the acidic region and the winged helix domain (WHD, Fig. 4f). Using yeast genetics, we show that these interfaces are relevant *in vivo*. Together, these data further refine recently published cross-linking data^19^,^20^. Furthermore, the mutants described here can be used *in vivo* to assess CAF-1 subunit contributions, without the need to deplete these proteins, hence minimizing secondary effects.

Previous studies using Cac3 homologues (human and *Drosophila*) in isolation suggested that the smallest CAF-1 subunit may be responsible for histone binding by CAF-1^21^,^22^,^23^,^24^. Using quantitative assays, we show that Cac3 has only minor contributions to H3-H4 binding within CAF-1. Apparently, in the context of this complex, the histone binding capacity of this subunit is not utilized as a primary interface. Nonetheless, our data suggest that Cac3 helps orchestrate the complex series of events that are required for the nucleosome assembly process, potentially by organizing the Cac1 central region. In fact, upon removal of Cac3, HX changes were observed throughout a ~115 residues region of Cac1, accounting for 19% of FL Cac1 residues and 30% of tCac1. It is possible that in absence of Cac3 this region becomes disordered, thereby interfering with the molecular mechanism of CAF-1 mediated chromatin assembly.

We show that the middle subunit Cac2 is required for productive histone binding, but only when in complex with Cac1, as it does not bind histones in isolation. This suggests that the Cac1-Cac2 interaction shapes the histone binding interface on CAF-1, which also includes the Cac1 acidic region, located in proximity to the Cac2 binding site. Our analysis demonstrates that in absence of Cac2 (tCAF_∆2), histones are bound in a non-productive form, as they exhibit a different HX protection pattern, and their assembly into tetrasomes is completely inhibited. It remains yet to be resolved whether Cac2 directly interacts with H3-H4 within CAF-1, or whether it plays a structural role that indirectly affects histone binding. Notably, the location of the Cac2-Cac1 interface places Cac2 at the nexus of the CAF-1 region responsible for histone deposition. Data from our lab^37^ shows that the Cac1 C-terminal WHD, which is a DNA binding motif^38^, coordinates nucleosome assembly, and its action is regulated by an intramolecular inhibitory interaction with the H3-H4 histone binding site, in particular the Cac1 acidic region. As the Cac2 binding site on Cac1 is located between these two Cac1 domains, it is plausible that Cac2 further controls the structural re-arrangements occurring on CAF-1 during the nucleosome assembly process. These data, together with the contribution of the middle subunit to the interaction with the histone chaperone ASF1^30^,^31^,^32^,^33^ places Cac2 at the heart of the histone binding activity of CAF-1 during chromatin assembly in DNA replication.

## Materials and Methods

### Cloning and reagents

cDNA for the three yeast CAF-1 subunits were a gift from Paul Kaufman. These were cloned into the MultiBac vector for expression in insect cells. Cac1 was cloned into pACEBac1, Cac3 in pIDC and Cac2 in pIDS and these were recombined by Cre-Lox as described^39^. A His-tag was inserted at the C-terminal end of Cac2 for purification purposes. All complexes are prepared with a short C-terminal deletion of Cac2 (1–449) to remove a region predicted to be highly disordered. Bacmids for expression in SF21 cells were prepared as previously described^39^. Mutations were introduced using Turbo Pfu polymerase (Roche) in a standard mutagenesis protocol. N-terminal sequencing was performed at the Protein Facility of Iowa State University. The result from the Edman degradation identify the Cac1 fragment starting with sequence FFKKLS. This corresponds to Cac1 residues 233–238. The structural models of Cac2 and Cac3 were generated using Phyre2 in intensive mode^40^.

### Protein purification

Histones were purified from *E. coli* cells as previously described^41^, and stored in 2 M NaCl at −80 °C. Labeling of histone proteins was performed as previously described^42^. We labeled H4 with AttoN-647 on T71C or E63C, and H2B with Alexa488 on T112C prior refolding with the histone partner. yCAF-1 was expressed in SF21 cells and purified using a HisTrap column (GE) in buffer containing 50 mM TRIS 8.0, 600 mM NaCl, 5% glycerol, 10 mM imidazole, 5 mM BME (beta-mercaptoethanol), in presence of COMPLETE EDTA-free, DNase I, 3 mM CaCl_2_ and 3 mM MgCl_2_. The complex was then loaded on a MonoQ column in buffer A (50 mM TRIS 8.0, 200 mM NaCl, 1 mM EDTA, 1 mM TCEP) and eluted with buffer B containing 1 M NaCl. The protein was then injected into a size exclusion column (Superdex 200) in buffer 30 mM TRIS pH 7.5, 300 mM NaCl, 1 mM EDTA, 1 mM TCEP. For HX-MS studies, buffer containing 50 mM KPO_4_, 150 mM NaCl, 5 mM DTT (pH 7.4) was used for the gel filtration step. Proteins were concentrated to 1–20 mg/ml and stored at −80 °C in their gel-filtration buffer. Mutants were purified as wild-type proteins. All mutant complexes were well-behaved and did not exhibit abnormalities during the purification procedure. The tCAF-1 complexes contain a deletion or mutation of the acidic region in Cac1 were purified over a MonoS, instead of the MonoQ column with buffer A and B at pH 6.8.

### Tetrasome assembly assays

The assays were carried out in buffer containing 25 mM TRIS pH 7.5, 150 mM NaCl, 1 mM EDTA, 0.02% Tween-20, 0.5 mM TCEP. CAF-1 was first diluted at different concentrations; normally a chaperone-histone ratio between 0.5 to 4 fold was used. Histones H3-H4 (100 nM tetramer concentration) were added, and the chaperone-histone mix was incubated at room temperature for 10 minutes. DNA was then added at 100 nM concentration. The reactions were incubated for 10–30 minutes (no differences were observed when incubating for longer time). Glycerol was added to a final concentration of 10% v/v prior loading of the samples on a 6% PAGE gel, pre-run in 0.2x TBE buffer at 4 °C. The gels were run for 70 minutes at 150 V at 4 °C. Gels were stained with SybrGOLD for 10 minutes and imaged on a Typhoon FLA 9500 at 488 nm.

### NAQ assay (Nucleosome assembly and quantification)

The assembly assay was carried out as described above containing 200 nM of 207 bp DNA, 200 nM (H3-H4)_2_, 400 nM H2A-H2B and titration of CAF-1 (100–200–400–800 nM). After the assembly reaction, the samples were diluted to a DNA concentration of 50 nM in 100 μl digestion reactions. 25 U of MNase enzyme was added in a final buffer containing 50 mM TRIS pH 7.9, 5 mM CaCl_2_. After incubation at 37 °C for 10 minutes, the reactions were quenched with 10 μl of 500 mM EDTA, pH 8. The DNA was then purified using a modified protocol of the MinElute kit from QIAGEN. 550 μl of PB buffer and 10 μl of 3 M sodium acetate were added to each sample and they were incubated at room temperature for 10 minutes. At this point, 50 ng of DNA loading control (or reference band, a 621 bp DNA fragment) was added to each tube. The samples were applied to the MinElute spin column and washed as prescribed by QIAGEN. The DNA was eluted with 10 μl of water. 1 μL of the eluate was used to load a DNA 1000 chip on the Bioanalyzer machine (Agilent), and 2.5 μl were loaded on a 10% PAGE gel. The gel was run for 45 minutes at 200 V in 0.5x TBE buffer at room temperature. Gels were stained with SybrGOLD for DNA and imaged on a Typhoon FLA 9500 (GE). The Bioanalyzer data was analyzed using the Agilent Expert 2100 software. The reference band was corrected for the proper size (621 bp) and the calculated molarity values were used to normalize all other bands present in the lane. The normalized values were used in the quantification and comparison. The signal threshold was set at 20 RFU. Nucleosome signal was calculated from bands ranging between 126–160 bp in length, based on the digestion of salt-reconstituted nucleosomes.

### Fluorescence Polarization experiments

Fluorescence Polarization assays were carried out in 25 mM TRIS pH 7.5, 300 mM NaCl, 5% glycerol, 1 mM EDTA, 0.01% NP-40, 0.01% CHAPS, 1 mM DTT (added fresh). Binding reactions were prepared by mixing 5 nM of Alexa488-labeled H3-H4 dimer and increasing amounts of CAF-1. Binding data were measured using a BioTek Synergy H2 plate reader. The data was analyzed and plotted using Microsoft Excel and GraphPad Prism.

### Size Exclusion Chromatography in line with Multi-Angle Light Scattering (SEC-MALS)

A Superdex 200 10/300 GL (GE) was mounted in line with a DAWN HELEOS II light scattering and a Optilab rEX refractive index detectors (Wyatt). The runs were performed in 20 mM TRIS pH 7.5, 300 mM NaCl, 1 mM EDTA, 1 mM TCEP at room temperature. 100 μL of protein sample at ~3 μM were injected at 0.3 ml/min, after being spun down at 14000 g for 10 minutes. Data analysis was done using the ASTRA software (Wyatt) and GraphPad Prism was used to prepare figures.

### Size Exclusion Chromatography in line with Small Angle X-ray Scattering (SEC-SAXS)

Superdex 200 (GE) 10/300 GL was mounted in line with the SAXS beamline at APS, Chicago. The runs were performed in 20 mM TRIS pH 8.0, 300 mM NaCl, 1 mM DTT at room temperature (25 °C). 500 uL of the FL CAF-1 complex was injected into the column at 1 mg/ml. Data was collected at APS, Beamline 18-ID-D with 1 sec exposures every 5 seconds, at 1.03 Å (12Kev). The camera length was ~3.5 m, the q-range we accessed was 0.0047 Å^−1^ to ~0.25 Å^−1^ and the detector used was a Mar165 CCD. Data reduction and buffer subtraction were done using the beamline-specific pipeline. The ATSAS software package was used for analysis^43^. After data analysis with primus, 10 independent ab initio models were generated with DAMMIF, with NDS values ranging between 0.483 to 0.695. These were then averaged with DAMAVER and DAMFILT generated the final model. The data shown here was generated based on the average curve between the five fractions coinciding to the highest absorbance/scattering during elution from the column. The results are very similar when each fraction was analyzed individually. GraphPad Prism and pdb2vol/VMD were used to prepare figures.

### Hydrogen Deuterium Exchange coupled to Mass Spectrometry (HX-MS)

HX was performed on stock samples of the CAF-1 complexes and/or histones at 4 μM in HX buffer (50 KPO_4_, 150 mM NaCl, 5 mM DTT pH 7.4). The CAF-1 complexes were gel filtered into HX buffer, and histones were dialyzed overnight in the HX buffer to ensure that the final samples would not contain any additional buffer components that may result in buffer variability between proteins. HX was initiated by mixing 5 μl of the 4 μM stocks with 45 μl of deuterated HX buffer (prepared by dissolving lyophilized HX buffer in 99.9% D_2_O) to result in an approximate final 90% D_2_O concentration. Exchange occurred for either 30 seconds, 1, 10, 30, or 60 minutes at 10 °C. Exchange was quenched by adding 50 μl of ice cold quench buffer (25 mM succinic acid, 25 mM citric acid at pH 2.2), that brought the reaction mixture to pH 2.4. Pre-quenched control reactions were prepared by adding quench buffer prior to D_2_O buffer. The samples were immediately injected into a temperature controlled (0  °C) Waters HDX Manager for online proteolysis at 12 °C using an immobilized pepsin column (Life Technologies), followed immediately by peptide trapping and an online 3 min desalting step at 0 °C using a Waters BEH UPLC C18 trap column for 3 min, all with 100% solvent A (0.1% formic acid in water) flow at 100 μL/min. Peptides were then separated at 0 °C using a Waters 100 mm BEH C18 analytical UPLC column and a linear 8% to 40% solvent B (0.1% formic acid in acetonitrile) gradient over 6 min, followed by a 1 min 40% B hold and subsequent ramp to 85% solvent B in 0.5 min using a Waters nanoAcquity UPLC and 40 μL/min flow rate. The UPLC was coupled directly to a Waters Synapt G2 HDMS q-TOF mass spectrometer operating in positive, MSe data acquisition mode. Samples were run and analyzed in a random order. Non deuterated, prequenched (indicated as 0.01 sec samples in the uptake plots), 1 and 60 minutes samples were taken in triplicates.

PLGS 3.0 (Waters) was used to create an identified peptide list from non-deuterated datasets and DynamX 3.0 (Waters) performed the search for deuterated peptide ion assignments. All isotope assignments for each peptide in each charge state were manually verified. The weighted average mass of each peptide determined by DynamX was then used to calculate deuteron uptake which was converted to % deuteration based on the number of maximum exchangeable amide protons. Data were corrected for artefactual in-exchange using the quenched experiment as previously reported^44^. No corrections for back-exchange were conducted due to the comparison of relative uptake amounts between bound and unbound states, which would remain unaffected by the back-exchange correction. Graph bars and uptake plots were prepared using Microsoft Excel and GraphPad prism. The uptake data of all the peptides analyzed in this study are shown in Supplementary Files 1 and 2.

### Yeast heterochromatin maintenance *in vivo* assay

The endogenous *CAC1* locus was cloned into a pRS313 vector (the fragment contained 659 bp upstream and 731 bp downstream the Cac1 ORF). An HA-tag was introduced in the 5′ end of the ORF. This construct was used to transform the PKY106 strain obtained from PD Kaufman^8^. Mutants were generated using site-directed mutagenesis and were treated as the WT sample. The empty pRS313 vector was used as control. Transformed PKY106 strains were grown in synthetic media lacking histidine (-His). Clones were amplified overnight and then diluted in the morning and grown to an OD600 = 0.7–0.8. After washing the cells with water and then resuspending them to an OD600 = 1, four 10-fold dilutions from the initial stock were prepared. The undiluted sample and these dilutions were spotted on plates containing -His or -His-Ura media as controls, and -His supplemented with 1 mg/ml 5-Fluoroorotic Acid (FOA) for growth selection. Plates were left at 30 °C for 2 days and then left at room temperature for up to the 7^th^ day. To validate HA-Cac1 expression, 7 ml of cultures were harvested at OD600 = 0.7–0.8 and washed in water. The pellet was boiled for 3 minutes. 50 μl of PBS buffer containing 1 mM TCEP and COMPLETE EDTA-free protease inhibitors was added to the pellet. Cells were lysed using glass beads and the lysate was then spun down. The supernatant was loaded on a SDS PAGE gel and transfer to a PVDF membrane. The blot was probed with anti-Cac1 antibodies (a gift from Zhiguo Zhang).

## Additional Information

**How to cite this article**: Mattiroli, F. *et al*. The Cac2 subunit is essential for productive histone binding and nucleosome assembly in CAF-1. *Sci. Rep.* **7**, 46274; doi: 10.1038/srep46274 (2017).

**Publisher's note:** Springer Nature remains neutral with regard to jurisdictional claims in published maps and institutional affiliations.

## Supplementary Material

Supplementary Figures

Supplementary File 1

Supplementary File 2

## Figures and Tables

**Table 1:** H3-H4 binding affinity and activity of tCAF-1 subcomplexes.

Complex	H3-H4 affinity – Kd (95% confidence intervals)	Tetrasome assembly	Nucleosome assembly
FL CAF-1	0.3 nM (0.27 to 0.45)	++	++
tCAF-1	0.5 nM (0.30 to 0.65)	++	++
tCAF_∆3	1.3 nM (0.91 to 1.79)	+	+
tCAF_∆2	25 nM (12.1 to 37)	—	—
Cac2	>1 μM	—	—
Cac3	77 nM (64.9 to 89.4)	—	—
HBM	1.9 nM (1.32 to 2.68)	N.D.	N.D.
tCAF_Cr∆3	>1 μM	N.D.	N.D.
tCAF_ Mr∆2	>1 μM	N.D.	N.D.
tCAF_ ∆ac	60 nM (41.9 to 78.8)	+	+
tCAF_ Nac	8 nM (6.16 to 9.29)	+	+
