# Supplementary material for: The Cac2 subunit is essential for productive histone binding and nucleosome assembly in CAF-1
Source: Sci Rep. 2017 Apr 18;7:46274. doi: 10.1038/srep46274 (PMC5394680; doi:10.1038/srep46274)
Supplement: Supplementary Figures [file srep46274-s1.pdf]

The Cac2 subunit is essential for productive histone binding and nucleosome assembly in CAF-1

Francesca Mattiroli[1,*], Yajie Gu[1,2,*], Jeremy L. Balsbaugh[3], Natalie G. Ahn[4], Karolin Luger[1,5]

[1]Howard Hughes Medical Institute, University of Colorado Boulder, Department of Chemistry and Biochemistry, Boulder, CO USA 80303. [2]Colorado State University, Department of Biochemistry and Molecular Biology, Fort Collins, CO USA 80523. [3]University of Colorado Boulder, Department of Chemistry and Biochemistry, Boulder, CO USA 80303. [4]University of Colorado Boulder, Biofrontiers Institute, Department of Chemistry and Biochemistry, Boulder, CO USA 80303. [5]Institute for Genome Architecture and Function, Colorado State University, Fort Collins, CO 80523.

* these authors contributed equally to this work

SUPPLEMENTARY FIGURES

Supplementary Figure 1   Mattiroli et al.

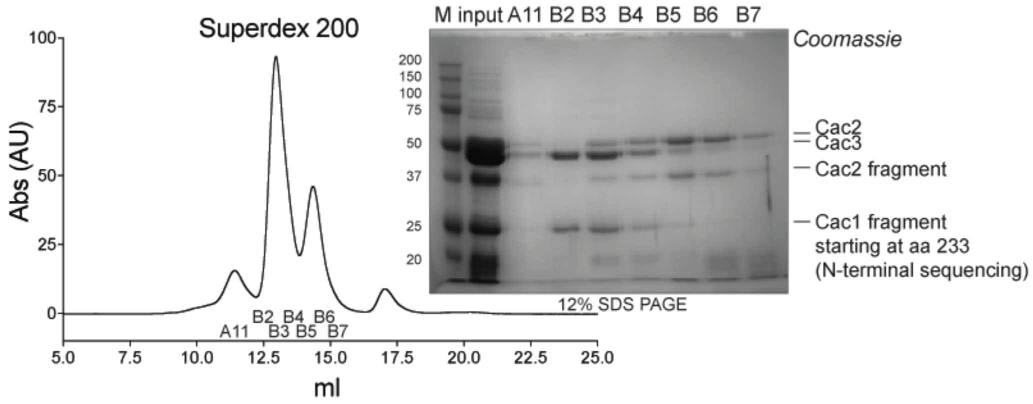

Supplementary Figure 1.

Size-exclusion chromatography trace (Abs 280 nm) of a degrading preparation of FL CAF-1. The injected sample and highlighted fractions are analyzed on a 12% SDS PAGE.

Supplementary Figure 2 Mattiroli et al.

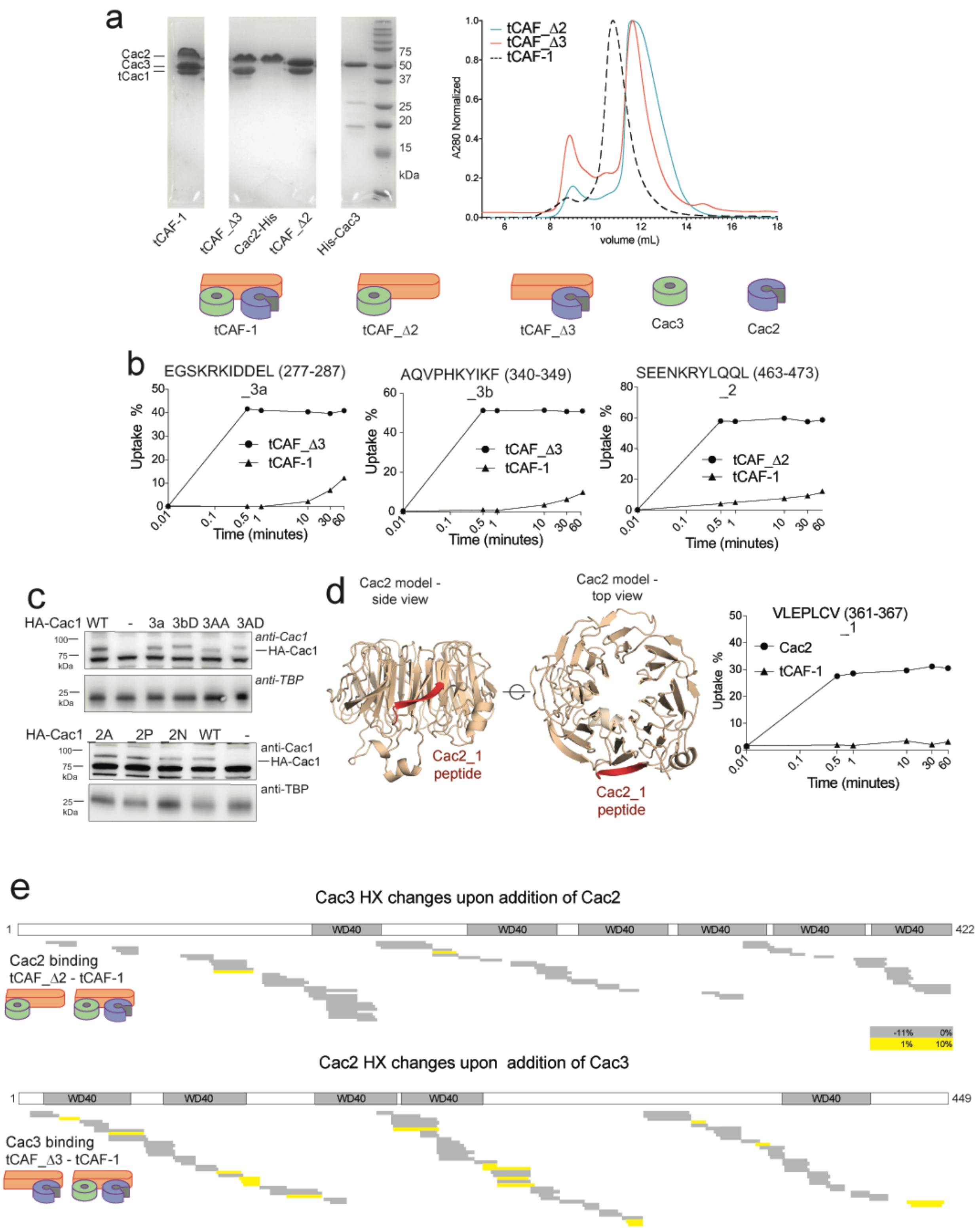

Supplementary Figure 2

a) Validation of the quality of tCAF-1, subcomplexes and subunits used in HX-MS. Left panel shows samples analyzed on 15% SDS-PAGE, full-length gel is shown in supplementary figure 3a; right panel shows gel filtration traces of tCAF-1 complexes. A280 absorbance is normalized for comparison; bottom panel shows a schematic of different complexes. **b)** Uptake plots of the Cac1 peptides mutated to test Cac2 and Cac3 binding. **c)** Western blot of the lysate of yeast strains used for the spot assay shown in Figure 2d, to verify expression of the Cac1 constructs. TBP was used as loading control. Full-length blots are shown in supplementary figure 3b. Cells were grown in minimal media without histidine to an OD600= 0.8. **d)** Structural model of Cac2 (calculated by Phyre2). The Cac2_1 peptide which binds to Cac1 is highlighted in red. Uptake plot from the HX-MS data of the Cac2_1 peptide. **e)** HX-MS heatmap of the differences in deuteron uptake at 60 minutes in Cac3 (above) and Cac2 (below) measured when probing their direct interaction. The difference was calculated as percent uptake in the subcomplexes (unbound form) minus the percent uptake in tCAF-1 (bound form). WD40 domain mapping in the schematics is based on the Uniprot prediction. Supplementary File 1 contains all the HX uptake values for the experiments shown in this figure.

Supplementary Figure 3 Mattiroli et al.

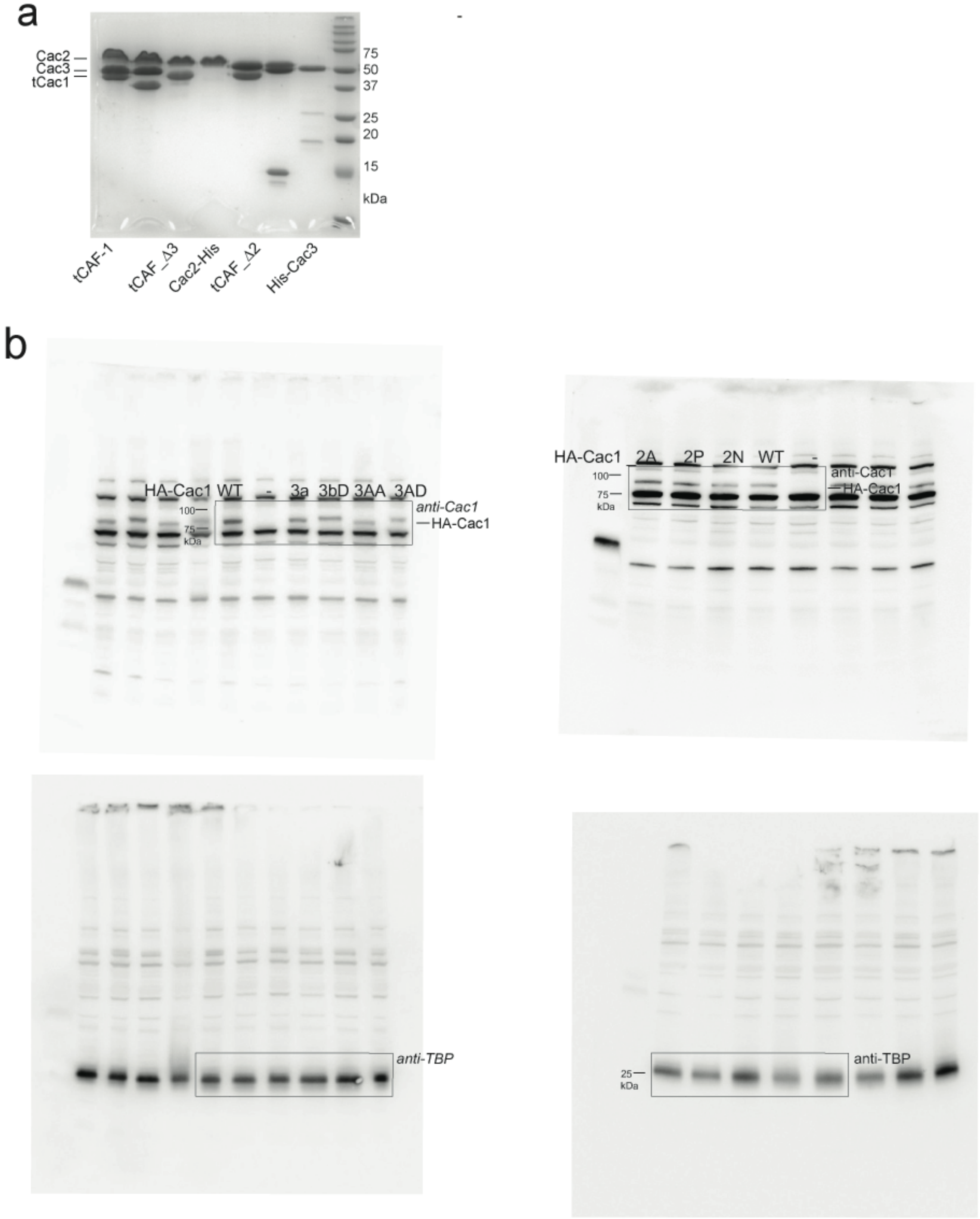

Supplementary Figure 3

a) full-length gel of the data presented in Supplementary Figure 2a. **b)** full-length blots of the data presented in Supplementary Figure 2c.

Supplemetary Figure 4 Mattiroli et al.

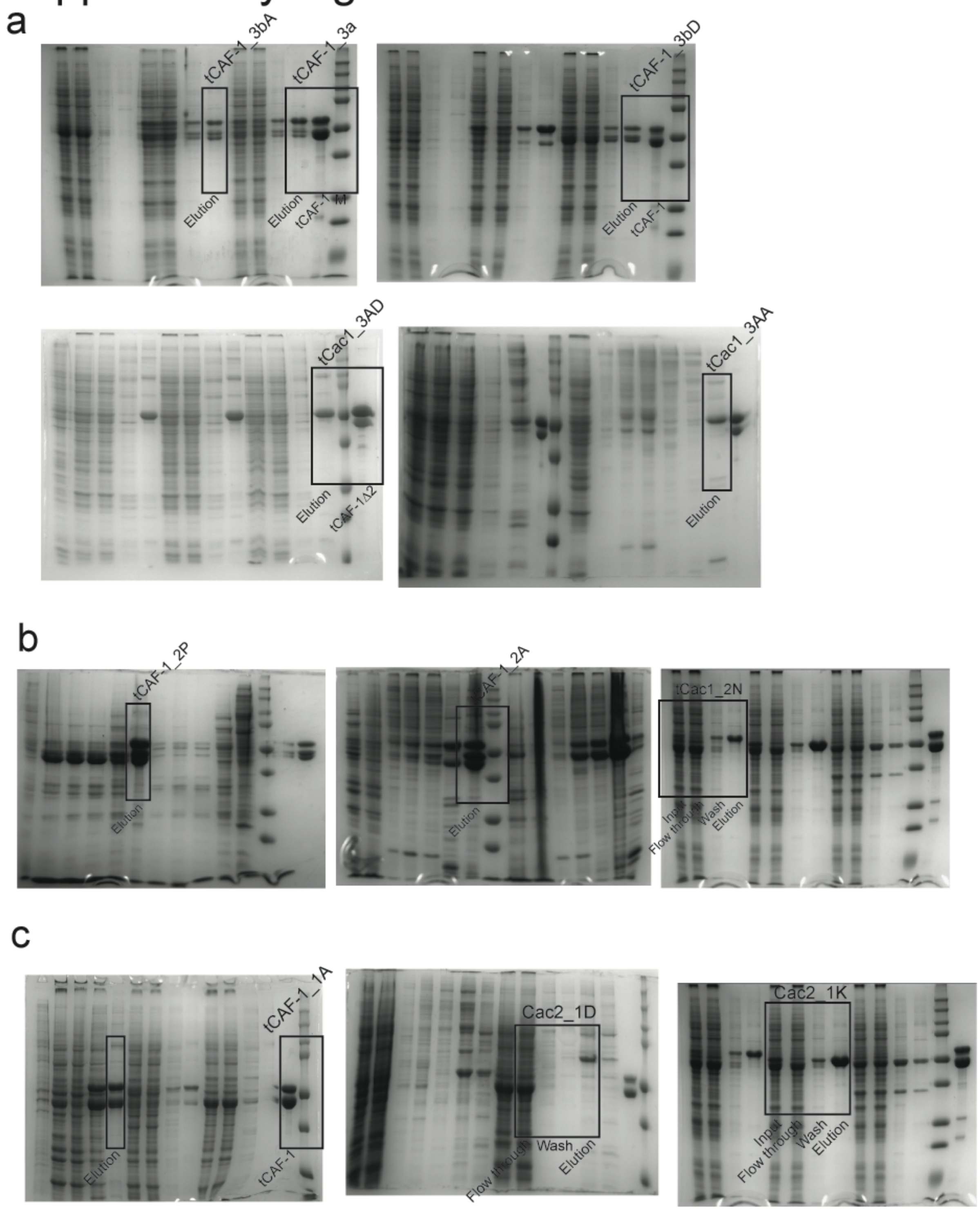

Supplementary Figure 4

a) full-length gel of the data presented in Figure 2b. **b)** full-length gel of the data presented in Figure 2c.

c) full-length blots of the data presented in Supplementary Figure 2g.

Supplementary Figure 5 Mattiroli et al.

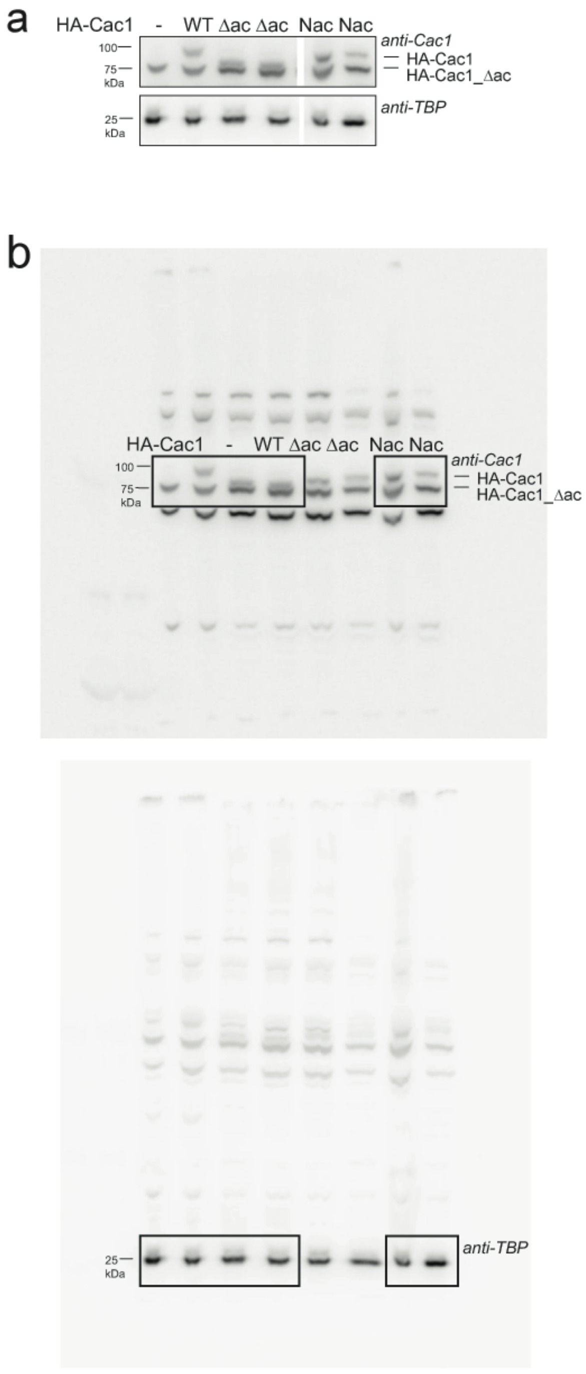

Supplementary Figure 5

a) Immunoblot analysis to confirm expression of Cac1_Δac and Cac1_Nac proteins in yeast cells used for the experiments shown in Figure 4e. The full-length blots are shown in panel b. **b)** full-length blots of the data presented in panel a.

SUPPLEMENTARY DATA FILES

Supplementary File 1: HX-MS data for all peptides analyzed in tCAF-1 subunits to study the interfaces involved in complex formation.

Supplementary File 2: HX-MS data for all peptides analyzed in wild-type H3-H4 in complex with tCAF-1 subcomplexes. The H3-H4 alone and tCAF-1•H3-H4 data are published in the related manuscript and are used here only as controls for the tCAF_Δ3•H3-H4 and tCAF_Δ2•H3-H4 samples.
